# 

**Published:** 1845-11

**Authors:** 


### NOTICE.

SUBSCRIBERS who have not complied with the published terms of subscription to the Medical Examiner, will oblige the publishers by remitting the amount due prior to the first of January, when the New Year will commence.

LINDSAY & BLAKISTON.

